# Clinical trial recruiters’ experiences working with trial eligibility criteria: results of an exploratory, cross-sectional, online survey in the UK

**DOI:** 10.1186/s13063-021-05723-6

**Published:** 2021-10-24

**Authors:** William J. Cragg, Kathryn McMahon, Jamie B. Oughton, Rachel Sigsworth, Christopher Taylor, Vicky Napp

**Affiliations:** Clinical Trials Research Unit, Leeds Institute of Clinical Trials Research, University of Leeds, Leeds, LS2 9JT UK

**Keywords:** Eligibility criteria, Inclusion criteria, Exclusion criteria, Recruitment, Protocol development, Generalisability, Quality assurance

## Abstract

**Background:**

Eligibility criteria are a fundamental element of clinical trial design, defining who can and who should not participate in a trial. Problems with the design or application of criteria are known to occur and pose risks to participants’ safety and trial integrity, sometimes also negatively impacting on trial recruitment and generalisability. We conducted a short, exploratory survey to gather evidence on UK recruiters’ experiences interpreting and applying eligibility criteria and their views on how criteria are communicated and developed.

**Methods:**

Our survey included topics informed by a wider programme of work at the Clinical Trials Research Unit, University of Leeds, on assuring eligibility criteria quality. Respondents were asked to answer based on all their trial experience, not only on experiences with our trials. The survey was disseminated to recruiters collaborating on trials run at our trials unit, and via other mailing lists and social media. The quantitative responses were descriptively analysed, with inductive analysis of free-text responses to identify themes.

**Results:**

A total of 823 eligible respondents participated. In total, 79% of respondents reported finding problems with eligibility criteria in some trials, and 9% in most trials. The main themes in the types of problems experienced were criteria clarity (67% of comments), feasibility (34%), and suitability (14%). In total, 27% of those reporting some level of problem said these problems had led to patients being incorrectly included in trials; 40% said they had led to incorrect exclusions. Most respondents (56%) reported accessing eligibility criteria mainly in the trial protocol. Most respondents (74%) supported the idea of recruiter review of eligibility criteria earlier in the protocol development process.

**Conclusions:**

Our survey corroborates other evidence about the existence of suboptimal trial eligibility criteria. Problems with clarity were the most often reported, but the number of comments on feasibility and suitability suggest some recruiters feel eligibility criteria and associated assessments can hinder recruitment to trials. Our proposal for more recruiter involvement in protocol development has strong support and some potential benefits, but questions remain about how best to implement this. We invite other trialists to consider our other suggestions for how to assure quality in trial eligibility criteria.

**Supplementary Information:**

The online version contains supplementary material available at 10.1186/s13063-021-05723-6.

## Administrative information

**Table:** 

**Title:**	Clinical trial recruiters’ experiences working with trial eligibility criteria: results of an exploratory, cross-sectional, online survey in the UK	
**Running Head:**	Recruiters’ experiences with eligibility criteria	
**Version + Date:**	Version 2.0 11-Oct-2021	
**Word count:**	4581	
**Tables/Figures:**	2 tables, 4 figures	
**Supplement:**	Eligibility criteria survey text Eligibility criteria survey invitation text CROSS checklist	
**Authors:**	**Name**	**Affiliation**
	William J Cragg w.cragg@leeds.ac.uk ORCID: 0000-0002-1274-8521	Clinical Trials Research Unit, Leeds Institute of Clinical Trials Research, University of Leeds, UK
	Kathryn McMahon	Clinical Trials Research Unit, Leeds Institute of Clinical Trials Research, University of Leeds, UK
	Jamie B Oughton ORCID: 0000-0002-2047-804X	Clinical Trials Research Unit, Leeds Institute of Clinical Trials Research, University of Leeds, UK
	Rachel Sigsworth	Clinical Trials Research Unit, Leeds Institute of Clinical Trials Research, University of Leeds, UK
	Christopher Taylor	Clinical Trials Research Unit, Leeds Institute of Clinical Trials Research, University of Leeds, UK
	Vicky Napp ORCID: 0000-0001-6726-2222	Clinical Trials Research Unit, Leeds Institute of Clinical Trials Research, University of Leeds, UK
**Correspondence:**	William Cragg w.cragg@leeds.ac.uk Tel: 0113 343 8398 Clinical Trials Research Unit Leeds Institute of Clinical Trials Research University of Leeds Leeds LS2 9JT, United Kingdom	
**Research support:**	This work was supported by a Cancer Research UK Core CTU infrastructure grant (reference A24929).	
**Summary of figures and tables**	Figure 1: characteristics of eligible survey respondents (*n* = 823) Figure 2: reported incidence of encountering problems with trial eligibility criteria. Figure 3: respondents’ reported primary method for accessing trial eligibility criteria Figure 4: respondents’ views on the possibility of earlier trial protocol review Table 1: Top 10 most frequent responses about types of problems experienced when using eligibility criteria Table 2: Top 10 most frequent additional comments about how eligibility criteria are developed or used	

## Background

Eligibility criteria, also known as inclusion and exclusion criteria, are a fundamental element of clinical trial design [1]. They define who can take part in the research, and who should not, thereby communicating who is expected to benefit from the trial intervention and who should not take part in the trial due to an unfavourable individual risk-benefit ratio [2]. They also indicate the extent to which a trial’s results may be generalisable outside the trial population and therefore whether the trial has an explanatory or pragmatic objective [3].

We suggest that optimal eligibility criteria are both (a) well-selected, in that they are collectively necessary and sufficient to help the trial achieve its objectives and protect patients in and outside the trial and (b) well-written, in that the intended meaning of each well-selected criterion is correctly and unambiguously conveyed to everyone who needs to understand it and, as far as possible, uniformly interpreted.

Suboptimal selection or writing of criteria poses risks to research quality and may reduce the ability of eligibility criteria to perform the roles outlined above. Misinterpretation of criteria leading to erroneous enrolment of participants who are not eligible can put those people’s safety and wellbeing at risk [4], or can undermine a trial’s integrity if many instances occur [5, 6]. Statistical challenges can arise both from the presence of ineligible patients in a trial cohort [7, 8], and from protocol amendments required to modify eligibility criteria partway through a trial [9]. Criteria that are designed, written or interpreted in ways that exclude a large proportion of people with the condition of interest may reduce the generalisability of a trial’s results [10] and may be unethical in denying people access to research participation and its associated benefits [11, 12]. Overly exclusive criteria may even mean trials exclude those most affected by a particular condition, as others have observed in context of the COVID-19 pandemic [13].

In multi-centre trials, there is a risk that different sites will interpret the eligibility criteria differently. Although this can be accounted for in the trial design through the common practice of using centre as a stratification factor in the randomisation [14], different interpretations of eligibility criteria are still undesirable. A trial’s eligibility criteria should be selected to achieve a balance between restrictiveness, to boost statistical power and protect patients, and permissiveness, to increase generalisability and facilitate recruitment [2, 15–17]. Site-level differences in interpretations represent deviations from this balance. If some sites interpret criteria too strictly, recruitment and generalisability may be negatively affected. If some interpret too loosely, eligibility-related protocol violations may occur, with the resulting statistical challenges and potential impact on patients’ safety mentioned above.

Clear communication of eligibility criteria is important in trial reporting, to aid reproducibility and the correct interpretation of trial results [18–20]. Problems in how criteria are written can also be an efficiency issue. Suboptimal criteria may take time to remedy through protocol amendments [21, 22] and may use up more staff time (both at trials units and at recruiting sites) in dealing with queries and uncertainty.

Problems with applying criteria are known to occur in practice [4, 5, 23–28]. Clearly, it can be difficult to foresee all potential problems, but we should nonetheless make all reasonable efforts to build in quality from the outset [29]. There is also an expectation from regulators that trial sponsors take all necessary action to prevent, and monitor the occurrence of, problems arising from eligibility criteria [30].

Eligibility criteria are typically chosen and written by members of a trial management group (led by the Chief Investigator) and included in a trial protocol. In multi-centre trials, the protocol is then shared with trial sites where recruiters—after all the required trial approvals are in place—use the eligibility criteria to assess patients for potential suitability for the trial. There is limited information about recruiters’ experiences interpreting and applying eligibility criteria, or about their views on how eligibility criteria are communicated and developed. We carried out a short, exploratory survey to gather new, primary evidence.

## Methods

We designed a short, cross-sectional, online survey to gather information on UK clinical trial recruiters’ experiences using eligibility criteria, as well as their views on how we might best communicate and collaborate with them. Given the risk of reduced response rate for longer surveys, we deliberately chose to include only a few key questions and to prioritise number of responses rather than information depth. The full survey text is available with the Supplementary Information to this article. We used Jisc Online Surveys [31] to host the survey.

Our choice of survey topics was informed by a wider programme of work underway at the Clinical Trials Research Unit (CTRU), University of Leeds, on how to assure the quality of trial eligibility criteria (i.e., to make sure they are well-chosen, well-written, and to minimise any risk of classification error [where patients who should be eligible are classified as ineligible or vice versa]). The CTRU is a well-established academic trials unit designing, conducting and analysing trials in a range of areas, such as trials of cancer treatments (including early phase trials [32]), surgical interventions and complex interventions. CTRU runs clinical trials with investigational medicinal products (CTIMPs) and those without (non-CTIMPs). Most CTRU trials are multi-centre, mainly recruiting in the UK but with some trials recruiting internationally.

The survey asked respondents about their trial experience in general, not only their experiences working on CTRU trials. It aimed to find out:

- How frequently recruiters encounter problems using eligibility criteria (to inform our understanding of the incidence of problems);
- For those who experience problems, the sorts of problems encountered (free-text description, with categorical questions about whether or not the encountered problems have led to patients being incorrectly included or excluded from trials);
- How eligibility criteria are typically accessed when needed, given that criteria may be available in more than one place (to inform our understanding of how recruiters access information and therefore where best to target any quality improvement efforts);
- The level of interest among recruiters for reviewing eligibility criteria during protocol development, i.e. when there is still a chance to influence the protocol content, as opposed to being presented with a final, approved protocol to implement (in our broader work, this was suggested as a way to improve eligibility criteria and we wanted to gauge interest in this).

The survey also provided space for any other comments about development or use of eligibility criteria.

Potential respondents were eligible for the survey only if they could answer positively to the first survey question: “Are you involved in assessing potential clinical trial participants against protocol eligibility criteria and are you currently working in the UK?”

We collected a limited amount of data about respondents’ characteristics (while maintaining individuals’ anonymity) to describe them and to enable exploration of any differences between respondent groups. Requested variables were as follows: medical doctor or not, levels of healthcare provided during career, experience working on CTIMPs and/or non-CTIMPs, number of years working on clinical trials and any experience writing trial eligibility criteria. We did not formally test the survey prior to using it, but did ask a recruiter to review it for clarity and appropriateness.

The survey was disseminated by email to professionals collaborating on trials run by the CTRU, with one reminder 2 weeks after the first notification. It was also disseminated through the National Institute for Health Research via relevant mailing lists, Twitter and a newsletter. The message accompanying the survey link (available in the Supplementary Information) said people could share it with other interested individuals. The survey was open between 8th August and 6th September 2019. In line with UK Health Research Authority guidance on proportionate consent [33], consent to participate was presumed to have been given when people chose to complete the survey. The software used to host the survey cannot prevent multiple participation, but we have no strong reason to suspect any individuals would have participated more than once.

Analysis of categorical data was descriptive only, presenting proportions (including for missing responses) with 95% confidence intervals for the population proportion. We also conducted exploratory subgroup analyses (not defined prior to data collection). As we were looking only to gather some basic information on this topic, we did not define a primary outcome and the survey had no pre-calculated sample size or statistical power (although before the survey we agreed that achieving at least 500 respondents would subjectively constitute success).

Responses to both of the comments fields were summarised via inductive analysis, working without a pre-existing framework to categorise each comment initially at a granular level (i.e. based on its specific contents) then combining these categories into broader themes. We chose this approach as we had neither a prior framework to work with, nor any strong rationale to make prior assumptions about the sorts of comments we would receive. For the comment field about types of problems experienced, coding was double-checked by another author (VN) for a random 10% of responses. All analysis was conducted by WC in Microsoft Excel.

We did not require ethical approval for this work, according to the Health Research Authority decision tool [34], and we did not collect any personal or confidential data. We have followed the best practice recommendations for reporting this sort of study and include a CROSS checklist with the Supplementary Information [35].

## Results

**Fig. 1:**
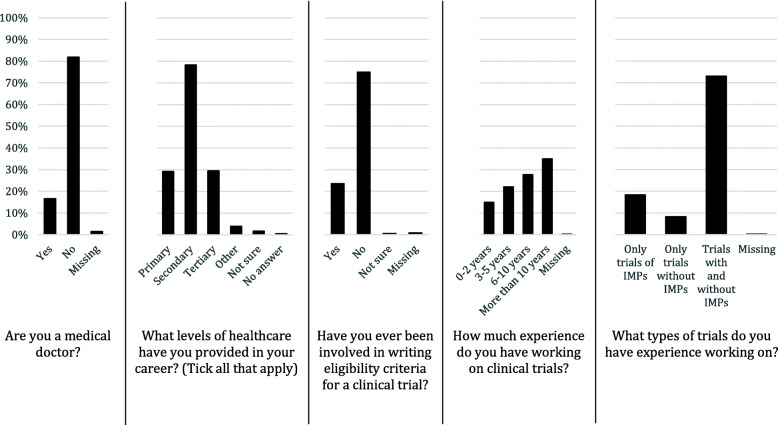
Characteristics of eligible survey respondents (*n* = 823)

A total of 823 eligible respondents took part in the survey (total responses: 874). Detailed information on their characteristics can be found in Fig. 1. One third of eligible respondents were not medical doctors, reported only experience in secondary care and had experience both of CTIMPs and non-CTIMPs.

**Fig. 2:**
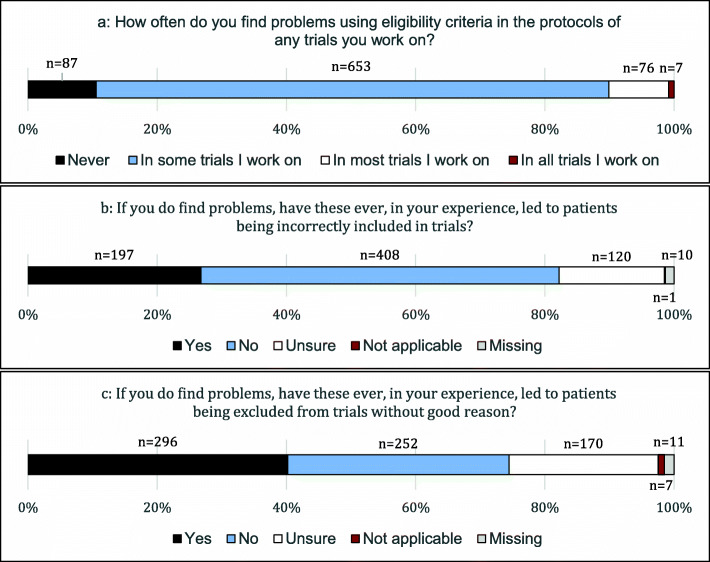
Reported incidence of encountering problems with trial eligibility criteria. **a** Incidence in general. **b** Frequency of incorrect inclusions. **c** Frequency of incorrect exclusions

Figure 2 summarises the responses to the quantitative survey questions about frequency of problems experienced when using eligibility criteria. In total, 653 respondents (79%, 95% confidence interval [CI] 77–82%) said that they find problems with eligibility criteria in some trials they work on, and a substantial minority (76 respondents; 9%, 95% CI 7–11%) said they find problems in most trials that they work on. Of the 671 respondents who commented about the most common types of problems, 448 (67%) mentioned issues of clarity (i.e. the meaning of eligibility criteria is not clear), 230 (34%) mentioned issues of feasibility (i.e. the meaning may be clear, but not achievable in practice, particularly due to required assessments within short timelines) and 91 (14%) mentioned issues of suitability (i.e. the meaning may be clear and processes may be feasible, but they disagree that criteria are necessary). Some respondents mentioned more than one theme within a single comment. Table 1 shows the top 10 most frequent responses to the free-text question about types of problems experienced.

**Table 1:** Top 10 most frequent responses about types of problems experienced when using eligibility criteria^a^

Rank	Comment type	Category (Clarity, Feasibility, Suitability, Other)	*n*	Proportion of all comments (*n* = 671)
1	Criteria generally ambiguous or unclear	Clarity	297	44%
2	Tests required in a short timeframe (i.e. difficult to achieve)	Feasibility	134	20%
3	Criteria too restrictive	Suitability	41	6%
4	Required tests not standardly done locally	Feasibility	40	6%
5	Hard to gather required data in time available (e.g. information hard to locate, stored in several places)	Feasibility	22	3%
6	Required timelines unclear	Clarity	20	3%
=7	Difficulty implementing subjective eligibility criteria	Clarity	19	3%
=7	Problems with wording, phrases or terminology used	Clarity	19	3%
9	Criteria too complex	Clarity	18	3%
10	Unclear which previous treatments allowed	Clarity	17	3%

^a^ Excluding comments that could not be categorised as the meaning was not totally clear, *n* = 33

Exploratory subgroup analyses of the question about frequency of problems showed little difference in responses depending on role (doctor vs non-doctor), or most aspects of experience (secondary care vs no experience in secondary care, experience in writing eligibility criteria vs none, years of trial experience). Respondents without CTIMP experience were more likely to say they had never experienced problems (29% [95% CI 19–40%] compared to 9% [7–11%] of those with CTIMP experience), a finding that did not seem to be explained by any of the other demographics variables.

Of those who said they find eligibility criteria problems in at least some trials, 197 (27%, 95% CI 24–30%) said these had led to incorrectly included patients and 408 (55%, 95% CI 52–59%) said they had not (18% unsure, missing or marked “not applicable”). By contrast, 296 (40%, 95% CI 37–44%) of the same respondents said problems had led to incorrectly excluded patients and 252 (34%, 95% CI 31-38%) said they had not (26% unsure, missing or marked “not applicable”).

**Fig. 3:**
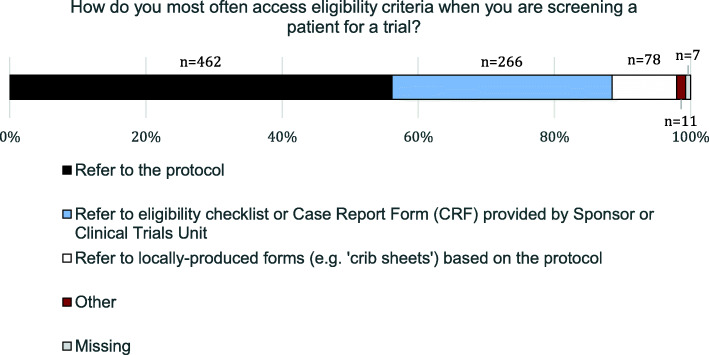
Respondents’ reported primary method for accessing trial eligibility criteria

Responses to the question about accessing eligibility criteria are shown in Fig. 3. A total of 462 respondents (56%, 95% CI 53–60%) said they access eligibility criteria by referring to the trial protocol. A second large group of respondents (32%, 95% CI 29–36%, *n* = 266) said their primary method was to use the sponsor-provided eligibility checklists or Case Report Forms (CRFs). Only 9% (95% CI 7–11%, *n* = 78) said they used locally produced forms based on the protocol (“crib sheets”).

**Fig. 4:**
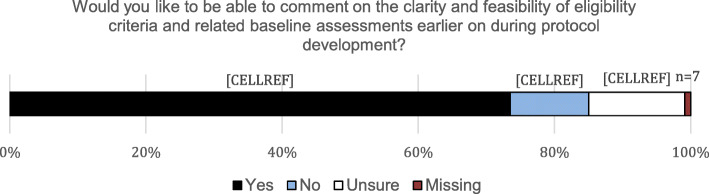
Respondents’ views on the possibility of earlier trial protocol review

Responses to the question about earlier review of eligibility criteria are shown in Fig. 4. A substantial majority of respondents (74%, 95% CI 70–77%, *n* = 605) said they would like to be able to comment on the clarity and feasibility of eligibility criteria and related baseline assessments earlier on in protocol development. Fifteen percent (*n* = 123) were unsure or did not respond to the question, and only 12% (*n* = 95) said they would not be interested in commenting on protocols earlier in development.

Table 2 shows the top 10 most frequent responses to the “other comments” free-text question. Of the 229 meaningful responses, the commonest themes were further views on problems experienced with eligibility criteria (69%, *n* = 157), further support for earlier review of trial protocols (34%, *n* = 78) and suggestions that current practice in developing trial protocols is already adequate (22%, *n* = 51).

**Table 2:** Top 10 most frequent additional comments about how eligibility criteria are developed or used^a^

Rank	Comment type	*n*	Proportion of all meaningful comments (*n* = 229)^b^
1	Support for earlier review of protocol/eligibility criteria	66	29%
2	Want clarity/consistency of information	20	9%
3	Already feedback to sponsor when criteria problematic	14	6%
4	Criteria are often too restrictive	12	5%
5	Criteria should be more inclusive of “real-world” patients	11	5%
6	Support for sponsor-provided eligibility checklists	9	4%
7	Happy to ask sponsor if have queries about criteria	8	3%
=8	Justification for criteria would be useful	7	3%
=8	Criteria can be long and complex	7	3%
=10	Criteria need to be more specific	6	3%
=10	Research Nurse involvement is/could be key in ensuring criteria quality	6	3%
=10	The right people/enough people already tend to be involved in protocol development	6	3%

^a^Full question: “Do you have any other comments about how eligibility criteria are developed or used?”

^b^Total responses: 282; excluded 51 for containing only “no comment” or similar; excluded 2 for comments on issues unrelated to eligibility criteria in trials

## Discussion

We conducted a simple survey to gather exploratory evidence on clinical trial recruiters’ experiences using eligibility criteria. Although we prioritised number of responses over detail, our survey suggests that recruiters often find challenges in implementing eligibility criteria. The comments explaining the sorts of problems that arise suggested that there are three main areas of concern: (1) clarity (unclear exactly what a criterion means), (2) feasibility (clear what a criterion means, but not easily achievable in practice, particularly with regard to required tests and timelines) and (3) appropriateness (clear what criterion means, and criterion and associated tests are achievable, but disagreement or uncertainty about why the criterion is necessary). Previous reports have raised the points on clarity [36] and feasibility [26], including the finding that as many as 7% of eligibility criteria in clinicaltrials.gov entries were “incomprehensible” [36]. Along with the previous evidence on the occurrence of eligibility classification errors and on unjustifiable exclusions, our results confirm that there is still work to do to improve eligibility criteria in trials. Our results also provide some evidence that there may be more problems in CTIMPs than non-CTIMPs, perhaps arising from the stricter regulatory environment in place in those sorts of trials.

The issue of clarity is perhaps the most likely to lead to classification errors, i.e. eligibility rules inadvertently being interpreted differently to the intentions of the protocol authors, leading to patients either being recruited when they should not have been, or incorrectly excluded on the basis of eligibility. Our survey does not directly provide data on how often errors of this kind occur, though data from other studies suggests they are not uncommon [5, 23–25]. These errors may or may not have a significant effect on trial integrity, but if small changes to how criteria are written could reduce error incidence and subsequently improve trial robustness and efficiency, these seem worthwhile. We should also not ignore the potentially significant impact that errors can have on individuals. Incorrect inclusions can put people’s safety at risk, if the breached eligibility criterion is in place to protect wellbeing. Incorrect exclusions can cause people inconvenience or upset, or unfairly deny them the potential benefits of research participation.

The issues of feasibility and appropriateness mentioned in free-text comments perhaps say more about exclusion than inclusion. At least some recruiters feel they are hindered by complex or demanding pre-trial procedures, or overly selective criteria. This is also borne out by recruiters more commonly experiencing patients being incorrectly excluded from trials than incorrectly included. This links to the recognised problem of limited generalisability of trial results, observed across various trial settings [10, 19, 37–42] and for at least the last few decades [17, 43]. Although other factors may contribute towards limited generalisability (such as recruiter discomfort in approaching some eligible patients [44] or underlying problems with the feasibility of interventions [45]), specific exclusions in eligibility criteria are likely to play a large part.

If the survey respondents’ impressions are correct, and incorrect exclusions are truly more common than incorrect inclusions, this may reflect risk aversion on the part of those designing and running trials (i.e. more efforts are made to prevent incorrect inclusions). Trialists might well be concerned about incorrect inclusions, as these can have implications for individuals’ safety in the short or medium term. However, in the longer term and therefore perhaps less immediately obvious, stricter criteria can have negative effects on trial results’ generalisability, trial recruitment [26, 46, 47] (still recognised as a key challenge in running successful trials [48–50]) and access to trial participation. Our work does not provide answers to how to find the correct balance between these competing priorities, and there may be no set of eligibility criteria that satisfies everyone. However, we suggest, as did some survey respondents and as have authors of previous reports [1, 2, 51, 52], that communicating the reasons for choices made in defining eligibility could at least give more transparency for recruiters (and even for potential trial participants [53]). We suggest this is useful for all criteria, even those that seem “self-explanatory” [51], for example in assessing the implications of eligibility classification errors during trial management or analysis (particularly where ineligible patients are entered into a trial). It may also be useful to justify the type of test or assessment for each eligibility criterion, especially where assessments are subjective [54]. If it is inconvenient to have this detail in the main body of the trial protocol, we suggest it could be available in an appendix instead.

The finding that recruiters most often said they referred to eligibility criteria in the protocol, closely followed by eligibility checklist CRFs, contradicted our prior assumption that many might refer to locally prepared “crib sheets” for participant recruitment. This is useful in knowing where best to target efforts for quality assurance and control. It is also helpful because the protocol and CRFs are under trial sponsor control, whereas locally prepared documents might contain errors or inconsistencies (or require additional sponsor or recruiter time in checking that there are no such errors). Others have suggested screening logs might also be a convenient place to list eligibility criteria [55]. Suitable quality control processes are needed to ensure all iterations of the criteria are complete and correct, within the protocol itself and in any other documents [30].

There was strong support among recruiters to have more involvement in reviewing protocols at a time when they could still influence the protocol content. Just as it is becoming ever more common to involve patients in trial design [56, 57], it also makes sense to consult, during the design phase, the people who will implement the trial protocol about its contents. This may already happen to some degree, and some survey respondents suggested this. However, it may currently be in a limited way, such as having a research nurse on the trial management group [58] (although in our experience, this is not always done). The group involved in developing a trial will usually include several clinicians, from separate healthcare centres. There may be an assumption that these clinicians’ standard practices and experiences are generalisable beyond their centres. Wider consultation at an earlier stage might helpfully scrutinise this assumption (including on issues like variation in normal lab values across trial centres [59]) and give greater reassurance about the chances of the trial recruiting to target and on time. It may also have benefits in terms of site training [60] or ensuring recruiters are comfortable with applying the eligibility criteria [44].

It is possible that this finding about earlier protocol review is particularly affected by the self-selected nature of the survey sample, i.e. people responding to our survey were more likely to approve of this than those who did not. We cannot discount the influence of selection bias; however, it does show that there is a group of recruiters who would be willing to carry out early reviews (even if this would not answer the further question of whether their views on the eligibility criteria are more broadly representative).

We accept that, in some settings, there may be no recruiters appointed or available during the protocol development stage. This would obviously preclude such an early review. However, in our experience it is reasonably common to have interested trial sites early on in trial setup, either through early engagement with potential recruiters about the new trial, or because the same sites were involved in previous, similar trials with the same sponsor.

Respondents’ comments highlighted some other potential challenges to carrying out this early review. For example, there were concerns about the possibility of accommodating different recruiters’ views, and scepticism about whether recruiters’ views could actually have any influence on protocol design. In particular, a few respondents were concerned about the time additional review would add to trial setup. We suggest a suitable mechanism is conceivable to overcome these barriers. For example, there could be a consultation period whereby a draft protocol is made available to all potential sites for a short period for feedback gathering. An organisation such as the UK Health Research Authority might be in a good position to facilitate such a process, which would be analogous to its pharmacy and radiation assurance schemes [61]. Although this might delay study setup times, it might be justified by reciprocal gains in terms of recruitment success or other efficiencies.

## Opportunities for improvement

Much of the published literature about eligibility criteria covers generalisability issues or “formalisation” of criteria for various informatics purposes [62, 63]. The latter group includes methods for using criteria in automated electronic health record data searching, either for finding potentially eligible trial participants [64, 65] or assessing trial feasibility [66–68].

There is relatively little prior evidence from a *quality* perspective, i.e. how to ensure criteria are well-selected, well-written, and not liable to misinterpretation or classification error [4, 5]. Some of the formalisation work could inform this topic. For example, the requirements for eligibility criteria to be clear and allow only binary responses apply in all settings, regardless of whether criteria are being evaluated by computers or humans. However, some criteria cannot easily be formalised for informatics purposes [69–71] and as long as these purposes are not common practice, we still need methods to assure quality in design and implementation of eligibility criteria in the context of use by human recruiters.

Prior reports about criteria quality suggest expert case review [6, 27], run-in periods [4] and an audit-feedback process [72] might be worthwhile interventions. However, it remains to be seen if any of these methods are scaleable or otherwise generalisable outside the settings they have been tried in, and the evidence supporting their effectiveness could not easily be described as robust.

We suggest all eligibility criteria should contain the same few core elements, namely a clear statement that allows only a “yes” or “no” response, a type of test or assessment (including where this may be subjective, or just checking existing data in medical notes) and a timeline for each trial-specific assessment (e.g. within *x* days of randomisation). For statements about past medical history, there should also be a timeframe, unless this is unambiguously implied (e.g. use of a gerund such as “breastfeeding” implies this is at the time of assessment). The test and timeline can be elsewhere in the protocol, but placing them beside or even within the eligibility criteria could ensure that these elements are present and that recruiters are fully aware of the requirements. Care should be taken with clarity of all time-related descriptions, particularly regarding exactly which “anchor” in time applies [63, 73, 74]. While publicly available protocol templates give general guidance on writing clear and complete eligibility criteria [75–77], we do not know of any that clearly state the need to include all the elements we have mentioned here. Although they may usually be included in practice, without clear guidance there is a risk they may sometimes be missed, with resulting negative impact on clarity or completeness of criteria.

We have also considered a review process to scrutinise criteria at the draft stage. This would include the review by potential sites, which our survey suggests has strong support from recruiters. It could also include targeted trial and data manager review, and Chief Investigator review to check the criteria are including and excluding the intended groups. Automated methods to compare criteria against those of similar trials may eventually help with this process [67] and this could also be a suitable time to check that the drafted criteria are suitably inclusive [13]. Our experience has been that implementing such a review is challenging, principally because of the lack of an optimal time to conduct it. Protocol development can be complex [78], with iterative drafting continuing until it is ready for its approval submissions, and at that point there can be little appetite to delay further. We therefore recommend building the various aspects of quality into the development process through training, templates and other such mechanisms. Clearly, all our suggestions would need further development and evaluation before being adopted more widely.

## Strengths and limitations

This was a brief, focussed survey with a substantial number of responses that gives weight to its conclusions. Our survey results may not be generalisable beyond UK academic trials, although from free-text comments it was clear that some survey respondents had experience working on commercially sponsored trials. Although survey respondents knew the overall results would be reviewed by the CTRU, individual responses were anonymous so we have no strong reason to suspect the questions were not answered honestly. The results give a clear message about the existence of suboptimal trial eligibility criteria, and willingness among recruiters to be involved in raising standards. The number of responses suggests considerable recruiter interest in this topic. We suggest the emerging themes from our work of clarity, feasibility and suitability may constitute a useful framework for evaluating other clinical trial processes.

We acknowledge several limitations not already mentioned. The survey was exploratory in nature, containing only a few questions because we consciously prioritised obtaining a larger volume of responses over more detail. Further work could collect more detailed data, and/or be statistically powered to answer a more specific research question. Our information on the responders’ characteristics could be considered limited, and due to the way the survey was disseminated, we are unable to give a precise survey response rate. Our survey does not provide evidence on the prevalence of classification errors in implementing eligibility criteria, but data on this is available elsewhere (see references already given). We also cannot easily comment on exactly why certain sorts of problems occur, but we suggest this could be a good subject for further research in this area.

The invitation message (see Supplementary Information) asked people to contribute even if they did not feel eligibility criteria were problematic. However, we cannot discount the possibility of selection bias in responses. There were no limits on the number of responses per site, so this may have affected the results in ways we cannot easily predict. However, respondents answered as individuals and there seems no strong reason to think that individuals in the same organisation would automatically have the same (or different) views.

Although we got recruiter feedback on the survey during its development, we did not formally validate our survey before use. There is therefore some chance that respondents interpreted the questions in varying ways, or that questions were inadvertently leading (despite our efforts to avoid this).

## Conclusions

The results of our exploratory survey confirm that, in this setting at least, problems for trial recruiters routinely arise from the content and clarity of trial eligibility criteria. These problems can have negative consequences both for trials and for individual patients. Recruiters strongly support the suggestion that they be more involved in protocol development at an earlier stage, although questions remain about exactly how to implement such involvement. Our finding that recruiters rely on sponsor-provided documents for accessing eligibility criteria helps sponsors target their quality assurance activity. We invite other trialists to consider our suggestions for how eligibility criteria should be developed.

## Supplementary Information

**Additional file 1.**

**Additional file 2.**

**Additional file 3.**

## Data Availability

The survey data supporting this work are available on reasonable request. Requests should be directed to the corresponding author in the first instance (w.cragg@leeds.ac.uk).

## Abbreviations

CI: Confidence interval
CRF: Case Report Form
CTIMP: Clinical Trial of an Investigational Medicinal Product (non-CTIMP: a trial without an IMP)
CTRU: Clinical Trials Research Unit (University of Leeds)
