# Correction to: Clinical trial recruiters’ experiences working with trial eligibility criteria: results of an exploratory, cross-sectional, online survey in the UK

**DOI:** 10.1186/s13063-021-05779-4

**Published:** 2021-11-29

**Authors:** William J. Cragg, Kathryn McMahon, Jamie B. Oughton, Rachel Sigsworth, Christopher Taylor, Vicky Napp

**Affiliations:** Clinical Trials Research Unit, Leeds Institute of Clinical Trials Research, University of Leeds, Leeds, LS2 9JT UK

**Correction to: Trials 22, 736 (2021)**

**https://doi.org/10.1186/s13063-021-05723-6**

Following the publication of the original article [1], we were notified that Figure 4 was not corrected as per the author’s request.

- Originally published Figure 4 
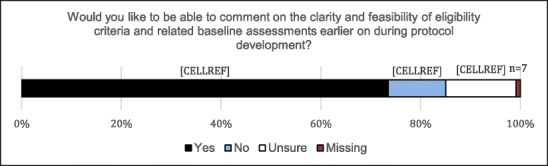
- Corrected Figure 4 
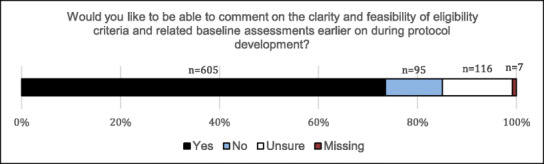

The original article has been corrected.

## References

[1] Cragg (2021). Clinical trial recruiters’ experiences working with trial eligibility criteria: results of an exploratory, cross-sectional, online survey in the UK. Trials.

