# Supplementary material for: Clinical trial recruiters’ experiences working with trial eligibility criteria: results of an exploratory, cross-sectional, online survey in the UK
Source: Trials. 2021 Oct 24;22:736. doi: 10.1186/s13063-021-05723-6 (PMC8542410; doi:10.1186/s13063-021-05723-6)
Supplement: Supplementary file 1 — Additional file 1. [file 13063_2021_5723_MOESM1_ESM.docx]

**Clinical trial eligibility criteria survey**

Thank you for your interest in this survey. We would like to find out more about your experiences using trial eligibility criteria.

There are only 6 short questions about the topic, with 5 additional questions to help us understand what sort of person has answered the survey. We expect the survey to take as little as **3-5 minutes** to complete.

We will use this to inform our protocol development processes at the Leeds Institute of Clinical Trials Research, University of Leeds [link: [www.leeds.ac.uk/LICTR](http://www.leeds.ac.uk/LICTR)]. We would also like to report results of the survey at the International Clinical Trials Methodology Conference 2019 [link: <https://ictmc2019.com/>] and in a peer-reviewed journal article.

This survey does not collect any personal data, and we expect all responses to be completely anonymous.

If you have any questions about this survey, please contact lead researcher Will Cragg at [w.cragg@leeds.ac.uk](mailto:w.cragg@leeds.ac.uk).

Terms used:

- **Eligibility criteria:** the list of conditions that must be met before patients are allowed to enter a trial. These can be inclusionary (patients **must** have X) or exclusionary (patients **must not** have X).
- **Baseline assessments:** tests carried out around the time that patients are considered for entry into a trial. Sometimes these link to the eligibility criteria, i.e. one or more criteria require specific tests to be done.

**Eligibility for this survey**

1. Are you involved in assessing potential clinical trial patients against protocol eligibility criteria **and** are you currently working in the UK?*

(*This could be in providing clinical opinions on a patient’s eligibility for a trial, gathering information to help assess eligibility, or some other involvement. However, do not complete this survey if you **only** complete Case Report Forms [CRFs] about eligibility or baseline assessments after patients have been recruited and have no other involvement in the process.)

- 1. Yes
  2. No [directed away from the survey if selected]

**Eligibility criteria questions**

1. How often do you find problems using eligibility criteria in the protocols of any trials you work on? (For example, unclear or ambiguous wording, mandated tests which are not easy for you to do within the given timeline, or other problems)
   1. Never
   2. In some trials I work on
   3. In most trials I work on
   4. In all trials I work on
2. If you do find problems, what are the most common? [Free text]
3. If you do find problems, have these ever, in your experience, led to patients being incorrectly **included** in trials?
4. Yes
5. No
6. Unsure
7. Not applicable
8. If you do find problems, have these ever, in your experience, led to patients being **excluded** from trials without good reason?
   1. Yes
   2. No
   3. Unsure
   4. Not applicable
9. How do you **most often access** eligibility criteria when you are screening a patient for a trial?
10. Refer to the protocol
11. Refer to eligibility checklist or Case Report Form (CRF) provided by Sponsor or Clinical Trials Unit
12. Refer to locally-produced forms (e.g. ‘crib sheets’) based on the protocol
13. Other [specify]

**For trials run by the CTRU in Leeds only:** if (c), we would be interested to see examples of these crib sheets to help inform our protocol and CRF development. If you would be willing to share, please send to [w.cragg@leeds.ac.uk](mailto:w.cragg@leeds.ac.uk). These will be handled separately to the survey data, and we will not link these to your answers to this survey.

1. In our experience, trial sites are usually sent protocols for local approvals once they are already finalised. There may therefore be not much scope for changing aspects such as the eligibility criteria if sites find them unclear or not feasible.

Would you like to be able to comment on the clarity and feasibility of eligibility criteria and related baseline assessments earlier on during protocol development?

1. Yes
2. No
3. Unsure
4. Do you have any other comments about how eligibility criteria are developed or used? [Free text]

**About you**

1. Are you a medical doctor?
   1. Yes
   2. No
2. What levels of healthcare have you provided in your career? (Tick all that apply)
   1. Primary
   2. Secondary
   3. Tertiary
   4. Other
   5. Not sure
3. Have you ever been involved in **writing** eligibility criteria for a clinical trial?
4. Yes
5. No
6. Not sure
7. How much experience do you have working on clinical trials?
8. 0-2 years
9. 3-5 years
10. 6-10 years
11. More than 10 years
12. What types of trials do you have experience working on? (Tick all that apply)
13. Trials of investigational medicinal products (CTIMPs, drug trials)
14. Trials not involving investigational medicinal products (non-CTIMPs, for example trials of devices or complex interventions)

**Final page**

Thank you very much for completing the survey. If you have any questions, or are interesting in hearing about the results of this work when they are ready, please contact the lead researcher Will Cragg at [w.cragg@leeds.ac.uk](mailto:w.cragg@leeds.ac.uk).
