# Supplementary material for: Clinical trial recruiters’ experiences working with trial eligibility criteria: results of an exploratory, cross-sectional, online survey in the UK
Source: Trials. 2021 Oct 24;22:736. doi: 10.1186/s13063-021-05723-6 (PMC8542410; doi:10.1186/s13063-021-05723-6)
Supplement: Supplementary file 2 — Additional file 2. [file 13063_2021_5723_MOESM2_ESM.docx]

**Invitation email**

**Subject: Invitation: 3-5 minute survey on trial eligibility criteria**

Dear colleague,

We are conducting a very short survey about eligibility criteria in trials. There are only a few questions and we believe you could complete this in as little as **3-5 minutes. <<<**Apologies if you have already received this invite through another route.>>>

Errors in applying eligibility criteria can lead to problems such as suitable patients being excluded from trials without good justification, or patients being put at risk by being enrolled when ineligible. Getting criteria right is vital for trial recruitment, appropriate generalisability of trial results and giving patients access to trial participation where they might benefit from it.

We would like to know if you ever find problems with how criteria are written (or, equally, if you don’t), how you use criteria in your routine practice, and how we might improve how we develop trial protocols. Your feedback on this issue would be very valuable in improving practices at the Leeds Institute of Clinical Trials Research and beyond.

The survey link is here: **[link]** It will close on **Friday 6^th^ September.**

All your responses are completely anonymous, so please feel free to be honest. We will use the results for developing our internal processes. We also intend to include a summary of the results in a peer-reviewed publication and in a poster at the International Clinical Trials Methodology Conference 2019 (<https://ictmc2019.com/>).

We hope you can help with this. Please feel free to pass to colleagues who do not work with CTRU trials but who might like to take part. More than one response per site is allowed.

This message has been sent to collaborators working with CTRU trials. We will send a reminder email in approximately 2 weeks’ time. If you wish to be excluded from this reminder, please let us know and we will ensure this wish is respected.

Best wishes,
